# App-Tailoring Requirements to Increase Stress Management Competencies Within Families: Cross-sectional Survey Study

**DOI:** 10.2196/26376

**Published:** 2021-07-30

**Authors:** Laura Luise Bischoff, Hannes Baumann, Charlotte Meixner, Patricia Nixon, Bettina Wollesen

**Affiliations:** 1 Department of Movement Science Faculty of Psychology and Human Movement University of Hamburg Hamburg Germany; 2 Fitbase Institute for Online Prevention GmbH Hamburg Germany; 3 Biopsychology and Neuroergonomics Technical University of Berlin Berlin Germany

**Keywords:** mhealth, ehealth, mobile applications, stress management, app features, gamification, family, personality traits

## Abstract

**Background:**

Families experiencing high levels of psychological distress are considered a particularly vulnerable population for adverse effects on mental and physical health. Moreover, highly stressed individuals engage less in mental health promoting activities and show low stress management competencies. App-based stress interventions seem promising for the treatment and prevention of stress outcomes and might be a low-threshold solution.

**Objective:**

The aim of this study was to identify the requirements for a tailored app to reduce stress in a cohort of highly stressed families that have low stress management skills.

**Methods:**

Parents (n=1008; age: mean 47.7 years, SD 6.1; female: 599/1008, 59.7%) completed an extensive web-based survey and were subdivided into a target (stressed individuals with low stress competency) and nontarget group according to their reported stress level and stress management competencies. Group differences were analyzed using analysis of variance. In principal component analysis with Kaiser varimax rotation, personally defined stress management goals were grouped into components. Linear regression models were also calculated.

**Results:**

A 3-factor solution cumulatively explained 56% of the variance in personally defined goals of interest for stress management with (1) active strategies (25.61% explained variance), (2) general competency (17.95% explained variance) and (3) passive strategies (12.45% explained variance). The groups differed in age (*F*_1,978_=27.67, *P*<.001), health index (*F*_1,958_=246.14, *P*<.001), personally defined general-competency goal (*F*_1,958_=94.16 *P*<.001), as well as “information acquisition” (*F*_1,971_=14.75, *P*<.001) and “need for stimulation” (*F*_1,981_=54.49, *P*<.001) personality traits. A regression model showed that for the active strategies goals of interest, only app feature information or instructional videos had a significant effect (*P*=.02). The general competency factor showed none, and the passive strategies factor showed significant effects for 2 app features—suggestions for planning possible activities with the family (*P*=.01) and diaries for documentation and development of strategies (*P*=.03).

**Conclusions:**

The results of this survey study highlight the need to develop an app to increase stress management competencies that takes into consideration perceived stress level, stress management skills, personality, and personally defined goals of the user. The content of the app should be tailored to previously detected personality traits, especially selective information acquisition and low need for stimulation. Furthermore, personally defined stress management goals seem to affect interest in some features.

## Introduction

### Background

Stress is associated with heightened risks of adverse physical and mental health consequences, such as impaired sleep [1], gastrointestinal diseases [2], diabetes [3], coronary heart disease [4], or depression [5]. These consequences are a tremendous burden from a societal, personal, and economical perspective. Families experiencing high levels of psychological distress are considered a vulnerable population [6]. Melchior et al [7] found, for example, that participants who are simultaneously exposed to elevated levels of work stress and high family demands have heightened rates of sickness absence due to psychiatric disorders. Studies investigating work–family spillover effects show that perceived stress at work can be transferred to family members [8-10]. In accordance with the work–family spillover theory, parents play a significant role in their children's health and coping by implementing or reinforcing certain behaviors [11,12]. Family stress was, for example, predictive of less adequate child dietary intake, with one effect occurring indirectly via impaired parent–child relationship quality [13]. In general, health is subject to sociostructural and milieu-specific dependencies for which the family is an important influencing factor [14-18]. It, therefore, seems to be of the utmost importance to create effective interventions to manage high stress levels in families.

### Stress Management Competencies

An increasing amount of literature suggests that interventions using different stress management techniques, such as mindfulness, lead to significant psychological health benefits in a wide range of populations [19-22]. Various stress management techniques have been applied and evaluated in diverse populations over the last decades in in-person settings or digital interventions. Active techniques, such as physical activity, can lead to a reduced perception of stress. Regular endurance and strength training, as well as yoga, have been shown to be effective in reducing stress [23-25] as well as acting as buffer against stress appraisal in times of elevated stress [25-27]. Similar results can be found for breathing exercises [28,29] and mindfulness training [30,31], with heterogeneous results for meditation exercises [32,33]. However, these active stress management techniques require regular practice. In comparison, passive but effective ways of managing stress and improving well-being are wellness and sauna [34] and spending time in nature [35,36].

In this study, we define the subjective ability to apply and perform such stress management techniques according to personal demands and stress level as *stress management competencies*. It should, however, be noted that highly stressed individuals are less likely to engage in mental health promoting activities [37-40]. Consequently, families reporting high stress levels presumably also have less stress management competencies. Thus, low-threshold options are needed (1) to support family members experiencing high levels of stress and (2) to teach stress management techniques.

### Tailoring in Mobile Health Stress Reduction Interventions

In an increasingly computer-educated European population, information and communication technology might provide unique and low-threshold opportunities to engage parents and families in mobile health (mHealth)–related services and encourage behavior change, to improve health and reduce stress [41-43].

New concepts, such as the PSYCHE system [44], have emerged as technology aids in order to improve or sustain mental health or stress monitoring [45]. Such wearables include personal health records and are designed to encourage health-related behaviors. Various mobile interventions with different guidance formats (eg, self-help, adherence-focused, eCoaching) have been developed to date and have been shown to be effective in the treatment of diabetes [46], depression [42,47], or sleep disorders [48]. Moreover, web-based stress management interventions seem promising for the treatment and prevention of detrimental stress-related outcomes [49]. Nevertheless, 2 meta-analyses [42,49] show that apps incorporating cognitive behavioral therapy or aspects of mindfulness training yield heterogeneous results. In fact, one of the biggest concerns about the usage of mobile interventions for health promotion is low adherence, which can be associated with reduced effectiveness [50,51]. For this reason, research has called for the examination of suitable components that could help to overcome this challenge.

Tailoring [52] was identified as having positive effects on the health outcomes of web-based interventions. Tailoring is defined as

any combination of information or change strategies intended to reach one specific person, based on characteristics that are unique to that person, related to the outcome of interest, and have been derived from an individual assessment. [53]

A meta-analysis on tailored print health behavior change interventions has demonstrated that tailored messages were superior compared to generic messages and were associated with larger effect sizes [52]. Moreover, variables such as gender or ethnicity did not moderate this effect which underlines the potential of tailored health communication to raise health-related awareness and knowledge about health for various target populations. To further capture the impact of tailoring, research has expanded to using the web as delivery mode, which again demonstrated the superiority against nontailored interventions [54,55]. Next to personalized messages, tailored web-based interventions often include gamification elements such as receiving rewards or social comparisons [56]. A comprehensive systematic review identified engagement promotion and enhancement of effectiveness as main reasons for the application of gamification [57]. Another systematic review on gamification demonstrated that, on average, only one gamification element, such as stories, themes, or display of progress was applied in web-based mental health interventions, with a maximum of 3 applied [56]. Altogether, these studies underline the vast opportunities for tailoring and the inclusion of gamification features and that users might perceive such interventions as more personally relevant and credible which again could have a significant impact on health outcomes.

Research on tailored web-based stress management interventions is scarce, yet tailoring could be an effective tool to empower users in their stress management skills and to reinforce self-determined health-related behaviors.

### Personality Traits

On the other hand, studies show that certain personality traits are associated with specific coping behaviors [58,59] and app usage behaviors [60,61], as well as the response to gamification elements [62]. Individuals with personality factors such as high neuroticism, for example, show more vulnerability toward high stress values and problem coping strategies such as wishful thinking, withdrawal, and emotion-focused coping [58,59]. Notably, personality might predict coping strategies in highly stressed samples more accurately than in less stressed samples [58]. Furthermore, conventional personality theories such as the Big Five Personality theory focus on cognitive or emotional contents to explain motivation and self-regulation. The Personality System Interaction (PSI) theory, on the other hand, focuses on:

functional relationships among affective and cognitive macrosystems, i.e., the dynamic processes that underlie human mental functioning [63]

and might be more suitable to detect and predict self-regulation and volitional aspects of health behavior. PSI theory distinguishes between 2 emotional—(1) the need for stimulation and (2) the need for security—and 2 cognitive systems—(1) the need for information and (2) information processing [64]. Meixner et al [65] investigated the associations between personality traits assessed via PSI theory, interest in app-based monitored physical activity goals, app features, and gamification in order to create tailored mHealth content and found no significant interaction. Furthermore, they concluded that the problem of inactive participants should, in fact, be addressed with app features and gamification elements in accordance with their prior defined goals rather than with their personality traits. Nevertheless, with respect to earlier studies suggesting personality traits being associated with higher stress vulnerability and potentially different stress management competencies, we hypothesize that the results by Meixner et al [65] might not be transferable to stress outcomes and tailored app features.

### Study Objectives

To develop and implement a tailored mobile app that appropriately reaches vulnerable und highly stressed families in order to improve their stress management competencies, the following aspects should be addressed: (1) existing stress management techniques and perceived stress management competencies in families, (2) the influence of personality traits, and (3) potentially suitable features for a mobile stress management intervention such as gamification and (4) defined goals of interest in order to individually manage stress.

Previous studies have focused on evaluation of the usage and tailoring effectiveness; however, evidence on the assessment of users' needs and preferences is limited. Given the adverse impact of low adherence on treatment outcomes, understanding technological and content-related factors is crucial for the design and large-scale implementation of app-based stress reduction interventions into routine health care, and ultimately, to help users to interact in a health-promoting way. With respect to the health impairing consequences of high stress levels for each family member, it seems highly relevant to evaluate the families' needs and preferences for mHealth approaches.

Therefore, the aim of this exploratory study was to identify the requirements of an individualized app to reduce stress in a cohort of highly stressed families that have low stress management skills.

The main research questions were (1) Which characteristics can be identified that describe stressed individuals with low stress competency? (2) Which app features and gamification elements are of the most interest for highly stressed participants with low stress-management competency? (3) Which app features and gamification elements are relevant for different types of stress management goals?

## Methods

### Study Design

This cross-sectional study was part of a project that aims to develop a tailored mHealth intervention for family members and to design health promotion in a sustainable manner. This study was approved by the University of Hamburg ethics committee (file reference: AZ: 2019_270).

### Sample

Every family insured by a small German health insurance cooperative (approximately 18,000 families) was invited by post to participate in a web-based survey. Participation in the study was voluntary, in accordance with the principles for medical research involving humans, and participation was not rewarded in any way. The questionnaire development process included several team-internal evaluation procedures and was implemented using Questback software [66], which allowed individual access via QR code. To avoid bias due to involuntary disclosure of sensitive information, there was a no disclosure option for each question. There were no mandatory questions for data protection reasons.

### Measures

The questionnaire is available as Multimedia Appendix 1.

#### Sociodemographic and Health Variables (8 items)

Age in years (1 item) and gender (1 item) were assessed. In order to provide a holistic framework, the concept *health behavior* was based on self-assessment in the dimensions of physical activity (2 items), dietary behavior (2 items), and stress (2 items).

For dietary behavior and physical activity, in each case, 2 questions from the CALO-RE taxonomy of behavior change [67] and the Baecke questionnaire [68] for measuring habitual physical activity were used in combination with the reference values of the World Health Organization [69] and the German Society for Nutrition [70].

A health behavior index was developed based on the physical activity, dietary behavior, and stress questions. For this purpose, each question was first evaluated on a scale of not achieved (0), partially achieved (1) and achieved (2) based on the reference values mentioned above. These values were added, resulting in a score between 0 and 12—if all questions were consistent with the proposed reference values in all 3 dimensions, a person reached an overall health index of 12. This means that the higher the health index, the more health-promoting a person's behavior.

#### Personally Defined Goals of Interest for Stress Management (10 items)

The following items were extracted from qualitative interviews: performance of meditation exercises, performance of breathing exercises, performance of yoga exercises, performance of mindfulness exercises, performance of relaxation exercises, improvement of stress management competencies, improvement of the ability to perform stress management techniques from anywhere, improvement of personal resilience to stress, spending time in nature, benefit from wellness and sauna offers. We first conducted interviews and then developed a quantitative survey with items extracted from the interviews. A query of the interest for these items was conducted using multiple checkboxes.

#### Personality Variables (16 items)

The personality questions were derived from previous qualitative interviews and checked for construct validity using the Visual Questionnaire [65]. The personality analysis included health-specific questions, which resulted in a manifestation of 4 personality dimensions (need for security, information acquisition, need for stimulation, and information processing). Each dimension was described by 4 items, each rated on a 6-point Likert scale that ranged from 1 (agree) to 6 (disagree).

#### App Feature Variables (9 items)

These variables were integrated into the web-based survey to identify tailoring requirements in accordance with our exploratory approach. The questions focused on preferences, ideas, and needs of the respondents in order to design an app in a user-friendly way that was adapted to their needs. The items were individualization of app content, fulfilling common weekly goals and tasks, connecting the app with wearables, increasing knowledge about a healthy lifestyle, suggestions for activities with the family, diaries for documentation and development of strategies, reminders of goals, informational or instructional videos, and analog format for children. Each item was rated by participants on a 6-point scale that ranged from 1 (totally irrelevant) to 6 (totally relevant).

#### Gamification Feature Variables (14 items)

Questions asking which gamification elements respondents found appealing—comparison with others, in a ranking or on a high score list; controlling and checking progress; collecting points for performance; collecting shared points with other family members; receiving awards, recognition, or encouragement; monetary incentives for achieving goals; linking to the bonus program of the health insurance company; designing an avatar; completing tasks under time pressure, for example, a countdown; advancing to another level or increasing the level of difficulty; sharing and comparing my achieved goals with others; an accompanying storyline; receiving auditory, haptic, or visual feedback; and rating other family members—were rated on a 6-point scale from 1 (would not appeal to me) to 6 (would appeal to me very much). Similarly, these were integrated into the web-based survey in an exploratory manner.

### Procedures

The web-based questionnaire (EFS Questback; 2019 version [66]) was preceded by participant information including instructions on anonymity, voluntariness, and data privacy. The participants received an invitation by post to complete the questionnaire. Completing the questionnaire took approximately 30 minutes. Only fully completed surveys were included in analysis.

### Statistical Analysis

We used SPSS software (version 27.0; IBM Corp) for statistical analyses.

#### Step 1

All variables of the questionnaire that asked for personally defined goals of interest for stress management were factor-analytically reduced to 3 factors (active strategies, general competency, passive strategies) in principal component analysis with Kaiser varimax rotation. Bartlett and Kaiser-Meyer-Olkin measure of sampling adequacy tests were performed to test the suitability of variables for factor analysis.

#### Step 2

Perceived stress level and stress-management competency variables were dichotomized in order to identify stressed individuals with low stress competency as a target group. The characteristics of the target group and the rest of the participants were descriptively characterized. We compared groups using analysis of variance.

#### Step 3

In order to analyze which app and gamification characteristics are relevant for stressed individuals with low stress competency, the data set was then reduced to only those participants, and to reduce data to relevant variables, all feature and gamification variables with a mean value <3.5 in the target group, indicating irrelevant features, or that did not differ significantly between groups were excluded.

#### Step 4

We performed 2-way correlation analysis between app and gamification feature variables not excluded in step 3 and the 3 stress reduction target factors (active strategies, general competencies, passive strategies) from step 1.

#### Step 5

Three linear regression models were calculated, each with 1 of the 3 target factors for stress management strategies (active strategies, general competencies, passive strategies) as a dependent variable. As independent variables, the remaining variables from step 3 (individualization of app content, fulfilling common weekly goals and tasks, increasing knowledge about a healthy lifestyle, suggestions for activities with the family, diaries for documentation and development of strategies, reminders for objectives, informational or instructional videos, and controlling and checking progress) were included.

### Power

In order to be able to demonstrate the anticipated small effect sizes (<0.05) in a multiple linear regression model with 95% power and 8 predictors, a minimum sample size of 463 was calculated (G*Power; version 3.1 [71]).

### Data Exclusion

Only fully completed questionnaires were included. For bi- and multivariate analysis procedures, list-wise case exclusion was used.

## Results

Of 18,000 families invited by post to participate in the web-based survey, 1008 families completed the questionnaire (a response rate of 17.86%). The total sample consisted of 599 female, 398 male, and 7 diverse participants; 4 participants did not give any gender information. The average age of respondents was 47.79 years (SD 6.13).

### Factor-Analytical Reduction of Personally Defined Goals of Interest for Stress Management

**Figure 1:**
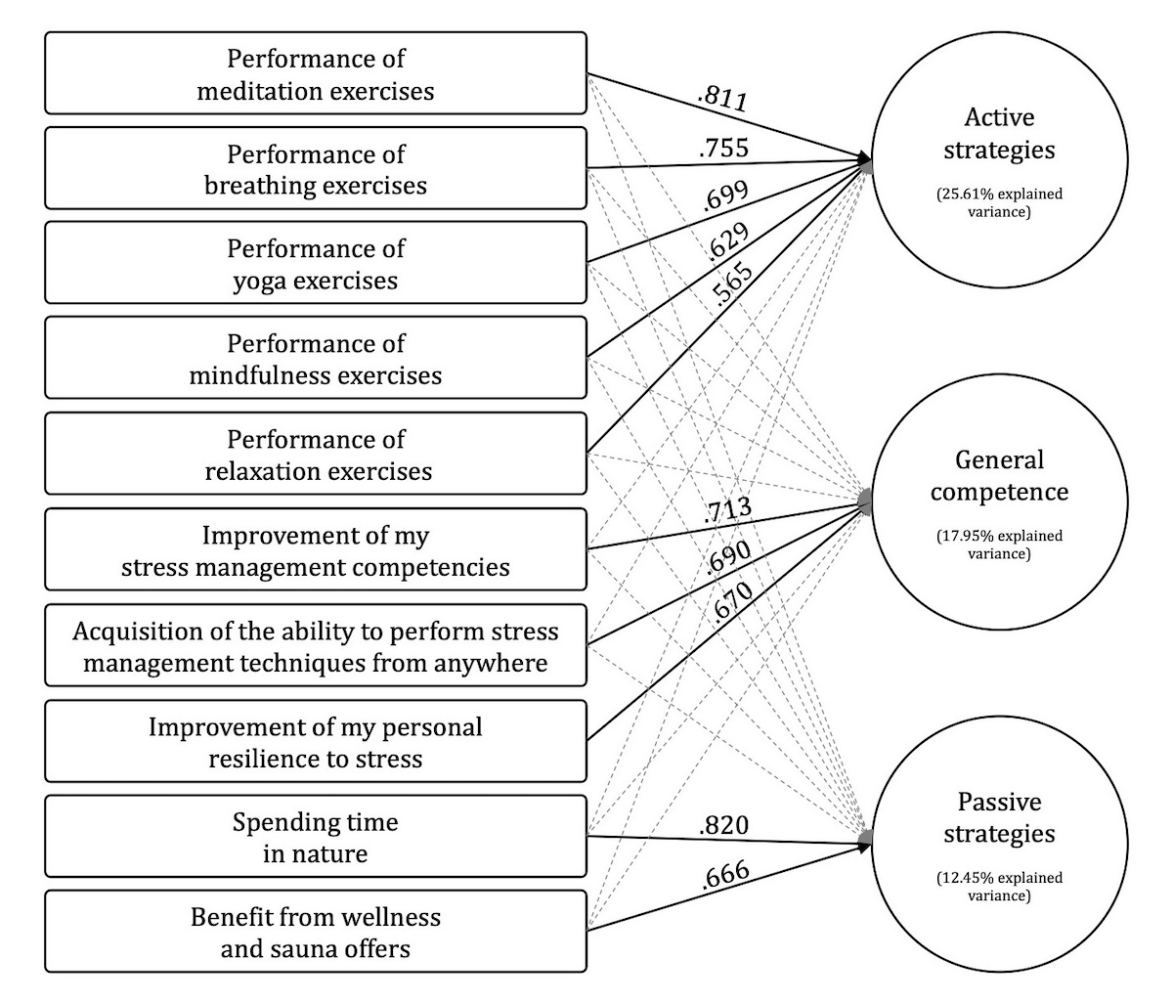
Rotated component matrix of the 10 stress-related target variables. Significant correlations are indicated by a continuous line.

Both the Bartlett test (χ^2^_45_=2105.563, *P*<.001) and measure of sampling adequacy (Kaiser-Meyer-Olkin .854) revealed that 10 stress-related target variables (Figure 1) were suitable for factor analysis. Principal component analysis, with varimax rotation indicated the presence of 2 factors with eigenvalues greater than 1.0, and a 3-factor solution that cumulatively explained 56% of the variance was chosen based on the scree plot (and theoretical considerations).

### Characteristics

A comparison of participants with low perceived stress, high stress management skills, or both versus participants with high perceived stress and low stress management (Table 1) demonstrated groups differed in age (*F*_1,978_=21.67, *P*<.001, ηp^2^=.022), health index (*F*_1,958_=246.14, *P*<.001, ηp^2^=.214), active strategies (*F*_1,958_=8.03, *P*=.01, ηp^2^=.008), general competency (*F*_1,958_=94.16 *P*<.001, ηp^2^=.086), information acquisition (F_1,971_=14.75, *P*<.001, ηp^2^=.053), and need for stimulation (*F*_1,981_=54.49, *P*<.001, ηp^2^=.012). App feature and gamification variables that met Step 3 criteria were individualization of app content (*F*_1,977_=8.95, *P*<.001, ηp^2^=.009), fulfilling common weekly goals and tasks (*F*_1,994_=7.80, *P*=.01, ηp^2^=.008), increasing knowledge about a healthy lifestyle (*F*_1,991_=9.06, *P*<.001, ηp^2^=.009), suggestions for activities with the family (*F*_1,993_=10.52, *P*<.001, ηp^2^=.010), diaries for documentation and development of strategies (*F*_1,990_=12.43, *P*<.001, ηp^2^=.012), reminders for objectives (*F*_1,995_=4.55, *P*=.03, ηp^2^=.005), informational or instructional videos” (*F*_1,994_=4.71, *P*=.03, ηp^2^=.005), and controlling and checking progress (*F*_1,998_=6.82, *P*=.01, ηp^2^=.007).

**Table 1:** Comparison of sociodemographic, personality, app feature, and gamification feature variables between groups.

Variables					Nontarget group (n=548)						Target group (n=460)							Comparison						
				n			Mean (SD)			n			Mean (SD)			*F* test *(df1,df2)*				*P* value			ηp^2^	
**Sociodemographic variables**																								
	Age (years)			534			48.62 (6.06)			446			46.81 (6.09)			21.67 (1,978)				<.001			.022	
	**Gender**																			— ^a^			—	
		Female	294			—			305			—			—									
		Male	250			—			148			—			—									
		Diverse	3			—			4			—			—									
	Health behavior index			525			6.64 (1.71)			435			5.06 (1.36)			246.14 (1,978)				<.001			.214	
**Personally defined goals for stress management**																								
	Active strategies			548			−.08 (0.04)			460			.09 (1.01)			8.03 (1,978)				.01			.008	
	General competency			548			−.26 (0.04)			460			.32 (0.87)			94.16 (1,978)				<.001			.086	
	Passive strategies			548			−.02 (0.04)			460			.03 (1.01)			0.537 (1,978)				.46			.001	
**Personality variables**																								
	Need for security			526			3.94 (0.83)			448			3.87 (0.83)			2.18 (1,972)				.14			.002	
	Information acquisition			525			3.66 (0.57)			448			3.52 (0.54)			14.75 (1,971)				<.001			.015	
	Need for stimulation			531			3.55 (0.62)			452			3.26 (0.61)			54.49 (1,971)				<.001			.053	
	Information processing			526			4.10 (0.59)			451			4.07 (0.58)			0.68 (1,975)				.41			.001	
**App feature variables**																								
	Individualization of app content			543			4.27 (1.76)			456			4.59 (1.57)			8.95 (1,978)				<.001			.009	
	Fulfilling common weekly goals and tasks			541			3.78 (1.58)			455			4.05 (1.48)			7.8 (1,978)				.01			.008	
	Connecting the app with wearables			534			3.02 (1.74)			448			3.38 (1.81)			10.07 (1,978)				<.001			.010	
	Increasing knowledge about a healthy lifestyle			538			4.09 (1.62)			455			4.38 (1.44)			9.06 (1,978)				<.001			.009	
	Suggestions for activities with the family			541			3.61 (1.61)			454			3.94 (1.53)			10.52 (1,978)				<.001			.010	
	Diaries for documentation and development of strategies			540			3.45 (1.57)			452			3.80 (1.51)			12.43 (1,978)				<.001			.012	
	Reminders for objectives			542			4.04 (1.58)			455			4.24 (1.47)			4.55 (1,978)				.03			.005	
	Informational or instructional videos			540			4.08 (1.60)			456			4.30 (1.47)			4.71 (1,978)				.03			.005	
	Analog format for children			548			1.72 (0.45)			460			1.68 (0.47)			2.56 (1,978)				.11			.003	
**Gamification** **feature variables**																								
	Comparison with others, in a ranking or on a high score list			544			2.26 (1.61)			454			2.26 (1.65)			0 (1,976)				.98			0	
	Controlling and checking my progress			546			4.25 (1.76)			454			4.53 (1.60)			6.82 (1,998)				.01			.007	
	Collecting points for my performance			545			3.60 (1.83)			455			3.79 (1.78)			2.66 (1,998)				.10			.003	
	Collecting shared points with other family members			541			3.50 (1.83)			453			3.59 (1.87)			0.61 (1,992)				.43			.001	
	Receiving awards, recognition, or encouragement			541			3.26 (1.77)			455			3.42 (1.76)			2.21 (1,994)				.14			.002	
	Providing monetary incentives for achieving goals			536			3.52 (1.88)			453			3.74 (1.81)			3.52 (1,987)				.06			.004	
	Linking to the bonus program of the health insurance company			541			4.16 (1.93)			456			4.40 (1.80)			3.97 (1,995)				.05			.004	
	Designing an avatar			541			2.46 (1.64)			454			2.72 (1.77)			5.69 (1,993)				.02			.006	
	Completing tasks under time pressure (eg, a countdown)			543			2.29 (1.50)			456			2.41 (1.58)			1.59 (1,997)				.21			.002	
	Advancing to another level or increasing the level of difficulty			542			3.22 (1.75)			457			3.39 (1.71)			2.38 (1,997)				.12			.002	
	Sharing and comparing my achieved goals with others			542			2.18 (1.47)			456			2.22 (1.50)			0.22 (1,996)				.64			0	
	An accompanying storyline			541			2.65 (1.70)			454			2.73 (1.64)			0.52 (1,993)				.47			.001	
	Receiving auditory, haptic, or visual feedback			539			3.06 (1.73)			455			3.27 (1.69)			3.9 (1,992)				.05			.004	
	Rating other family members			539			2.43 (1.60)			454			2.46 (1.62)			0.05 (1,991)				.82			0	

^a^Data not provided.

### Correlations Between Personally Defined Goals of Interest for Stress Management and App Features in the Target Group

The personally defined *active strategies* factor was correlated with 5 of the 8 features (increasing knowledge about a healthy lifestyle, suggestions for activities with the family, diaries for documentation and development of strategies, reminders for objectives, and informational or instructional videos) (Table 2). The *general competency* factor was correlated with fulfilling common weekly goals and tasks, diaries for documentation and development of strategies, and reminders for objectives. The *passive strategies* factor showed the lowest correlations with the features; it was only correlated with suggestions for activities with the family and diaries for documentation and development of strategies. While some features were correlated with several target factors, others were specific to one factor.

**Table 2:** Correlations between personally defined goals and app features in stressed individuals with low stress competency.

Feature variables^a^	Active strategies				General competency				Passive strategies		
	*r*	*P* value	n	*r*		*P* value	n	*r*		*P* value	n
Individualization of app content	0.044	.35	456	0.037		.43	456	0.076		.11	456
Fulfilling common weekly goals and tasks	0.086	.07	455	0.107		.02	455	0.068		.15	455
Increasing knowledge about a healthy lifestyle	0.143	.002	455	0.066		.16	455	0.081		.08	455
Suggestions for activities with the family	0.158	.001	454	0.084		.07	454	0.152		.001	454
Diaries for documentation and development of strategies	0.136	.004	452	0.104		.03	452	0.125		.008	452
Reminders for objectives	0.145	.002	455	0.135		.004	455	0.048		.30	455
Informational or instructional videos	0.201	<.001	456	0.090		.05	456	0.061		.19	456
Controlling and checking progress	0.030	.52	454	0.034		.47	454	0.005		.92	454

^a^All significant correlations were considered to have a small effect.

### Integration of the Feature Variables in Linear Regression Models

We found that the correlations were partially eliminated in multivariate models (Table 3). For the *active strategies* factor, only information or instructional videos had a significant effect (*P*=.02). The *general competency* factor showed none, and the *passive strategies* factor showed a significant effect for suggestions for planning possible activities with the family (*P*=.01) and diaries for documentation and development of strategies (*P*=.03).

**Table 3:** Integration of the feature variables in 3 linear regression models.

Feature variables	Active strategies			General competency			Passive strategies	
	β	*P* value	β		*P* value	β		*P* value
Individualization of app content	−.085	.21	−.042		.54	.013		.85
Fulfilling common weekly goals and tasks	.003	.97	.055		.47	.025		.74
Increasing knowledge about a healthy lifestyle	.022	.75	−.008		.91	−.026		.70
Suggestions for activities with the family	.085	.20	.005		.94	.174		.01
Diaries for documentation and development of strategies	.051	.45	.014		.84	.149		.03
Reminders for objectives	.063	.46	.152		.08	−.129		.13
Informational or instructional videos	.154	.02	.023		.73	.023		.73
Monitoring and checking progress	−.099	.11	−.086		.18	−.072		.25

## Discussion

### Principal Findings

The main goal of this cross-sectional study was to identify the requirements for an app that addresses stress management competencies in a cohort of highly stressed family members. We analyzed the characteristics of the target group, their individualized interests in app features and gamification aspects, their personality traits, and different types of personally defined goals.

Almost half of the study sample was identified as the high-risk population—stressed individuals with low stress competency. This underlines the importance of this study's aim. Furthermore, this group's size reflects the ever-increasing proportion of people who feel unable to effectively cope with stressors in their everyday and work–life situations, which is why the World Health Organization classified stress as the health epidemic of the 21st century and called for prevention strategies [72]. As expected, further analysis of the target group revealed a lower health index—a marker for individual health behavior based on physical activity, dietary behavior, and stress management—than that of the group with lower perceived stress levels. This finding is in line with those of prior studies, indicating that highly stressed individuals are less likely to engage in mental health promoting activities [37-40].

Notably, the target group also differed in their personally defined goals of interest for stress management. The parents who stated that they experience high stress and have low stress management competency aimed to achieve general competencies such as improvement of stress management competencies, acquisition of the ability to perform stress management techniques from anywhere, and improvement of personal resilience to stress. The nontarget group, however, aimed to achieve active strategies including performance of yoga exercises. This highlights the need to differentiate between the groups when developing and implementing mobile solutions to improve stress management competency. According to our results, the target group did not formulate specific goals but tended to have unspecific, general goals. Therefore, one might speculate that participants with subjectively higher stress levels and lower stress management competencies need more help with goal setting and more information about which strategies might reduce stress. These results further emphasize the need for tailored app features for highly stressed families. In line with Control Theory, behavior change techniques, such as goal setting, have been associated with increased intervention effects [73,74]. A study [75] evaluated a newly developed internet-based stress management intervention in a waitlist-controlled randomized trial that included principles for health behavior change such as goal setting, action planning, and coping planning for reducing stress in employees with elevated stress levels; their results showed significantly large effect differences between the intervention and waitlist control group for perceived stress at posttest. Goal-setting techniques features might thus be promising for the individual needs of stressed individuals with low stress competency.

The analysis of specific app features revealed further differences between the 2 groups regarding their app-related interests. The target participants, with low stress management competencies, indeed showed higher ratings for app features that can be used as goal-setting techniques: weekly goal and task achievements, diaries for documentation, and development of strategies and reminders for objectives. Furthermore, they had higher ratings for content individualization, connecting wearables to the app, increasing knowledge about a healthy lifestyle, suggestions for activities with the family, and informational or instructional videos. Overall, these results indicate that users identified as the high-risk population, with low stress-management competencies and high perceived stress levels, wish for features that facilitate the usage of the stress management apps. These requested features can be primarily interpreted as a need for coaching and instruction. In fact, such guided interventions have been shown to be more effective compared with unguided interventions [76]. Moreover, studies suggest guidance is conducive to the effectiveness of stress management interventions [49]. The support that might be provided in eHealth interventions can be technical or content-related, in order to ensure the correct usage [77].

A comprehensive systematic review [57] has established engagement promotion and enhancement of effectiveness as main reasons for the use of gamification in mental health promotion. In an attempt to meet this call, our study investigated the participant’s interest in such elements. Nevertheless, among gamification feature variables, only controlling and checking progress met relevance criteria. This leads to the assumption that the interest in gamification elements is mostly independent of perceived stress competencies and stress levels. The greater interest of the target group in tracking features of their progress further underpins the assumed need for coaching and instruction as well as goal setting.

Interestingly, persons reporting high stress levels and low stress management skills were younger than participants with lower self-reported stress and differed significantly in 2 personality traits variables. Specifically, their personality structure indicated lower scores in information acquisition (*P*<.001) and need for stimulation (*P*<.001). According to PSI theory, stressed individuals with low stress competency thus have more selective information acquisition and lower needs for stimulation than the nontarget group members [64]. Kuhl [78] describes individuals with selective information acquisition in accordance with Jung [79] by pointing out their analytical and structured thinking and their intuitive ability to control their behavior. Unconscious perception and behavior programs may consequently support them. A low need for stimulation, in contrast, indicates less action-oriented and more introverted behavior [64]. This is frequently associated, in other studies [80,81], with the occurrence of anxiety, stress, and depression. Thus, our results suggest that these 2 personality traits are vital for the perception and management of stress. Literature repeatedly demonstrated this influence of personality traits on stress perception [82,83]. For the development of an app to improve stress management skills, individualization of the content based on personality structure will address individuals in the target group more adequately. The content should be tailored to people with selective information acquisition and a low need for stimulation. This might be achieved by a minimalistic user interface, decreasing the participants' stimulation, thereby focusing their attention on the essential contents. Ervasti et al [84] were able to show, with a study on the influence of personality on interest in stress apps that high neuroticism levels (originated in the Big Five theory, but conceptually comparable to low stimulation need in PSI theory) were positively correlated with interest in stress management apps, which is analogous to the results of this study. The predictive value of neuroticism on stress perception was also highlighted in a comprehensive meta-analysis [58].

Furthermore, it appears that interest in some features was higher, depending on specific personally defined goals of interest*.* While multiple correlations were found between personally defined goals and app features, correlations were partially eliminated in multivariate models. Nevertheless, our results indicate that the probability of interest in informational or instructional videos was higher when active goals had been set. Similarly, interest in suggestions for family activities and diaries was higher when personally defined goals yielded passive strategies.

Our results support the theory that creating a stress management app requires tailored content to address the differences in perceived stress levels, stress competencies, and personality traits. These findings build on those of Lustria et al [54], who pointed out that presenting general health information without considering individual needs or personal relevance may substantially limit the extent of health behavior change. Our study substantiate this call for individualized messaging based on preassessment of key individual-difference variables by reinforcing the notion that highly vulnerable families with low perceived stress competency need an individualized app content, additionally tailoring different personality types. Tailoring works by increasing the personal relevance of health messages [85] and holds promise.

### Limitations and Future Research Directions

One limitation of the study is that only individual parents were surveyed. Assessing more than one family member could not be realized at this stage of the project but will be aimed for in future studies. With respect to spill-over effects of perceived stress across family members [8-10], a holistic analysis of requirements of an app that targets the entire family is needed.

A further limitation is that the identification of our target group was only based on 2 items. This was due to practical reasons and the length of the existing survey in a larger research project. The results presented and discussed in this paper can, thus, only be regarded as exploratory and should be replicated using validated scales such as the Perceived Stress Scale [86]. Nevertheless, because of the large sample size, our results underpin those from existing research, reinforcing the notion of tailoring in the development of web-based stress management interventions.

Future research should bring these preliminary data into practice and develop and evaluate an app that adequately addresses the stress level and stress competency as well as personality traits and personal goals of the user. Furthermore, there is still no evidence to support whether already existing apps are well accepted by our target group and whether these apps provide motivating factors for long-term use to build and maintain stress management skills. For this reason, further studies on the sustainable development of apps and the support of behavior change processes, in the course of stress management, should identify the situations and conditions that can have an impact on work–life stress, coping, and goals of individual family members.

There is also a need for further research in this area to provide sustainable evidence for features and gamification elements with the aim of developing age- and gender-specific stress coping skills and, if necessary, to enable transfer to other digital media.

### Practical Implications

**Figure 2:**
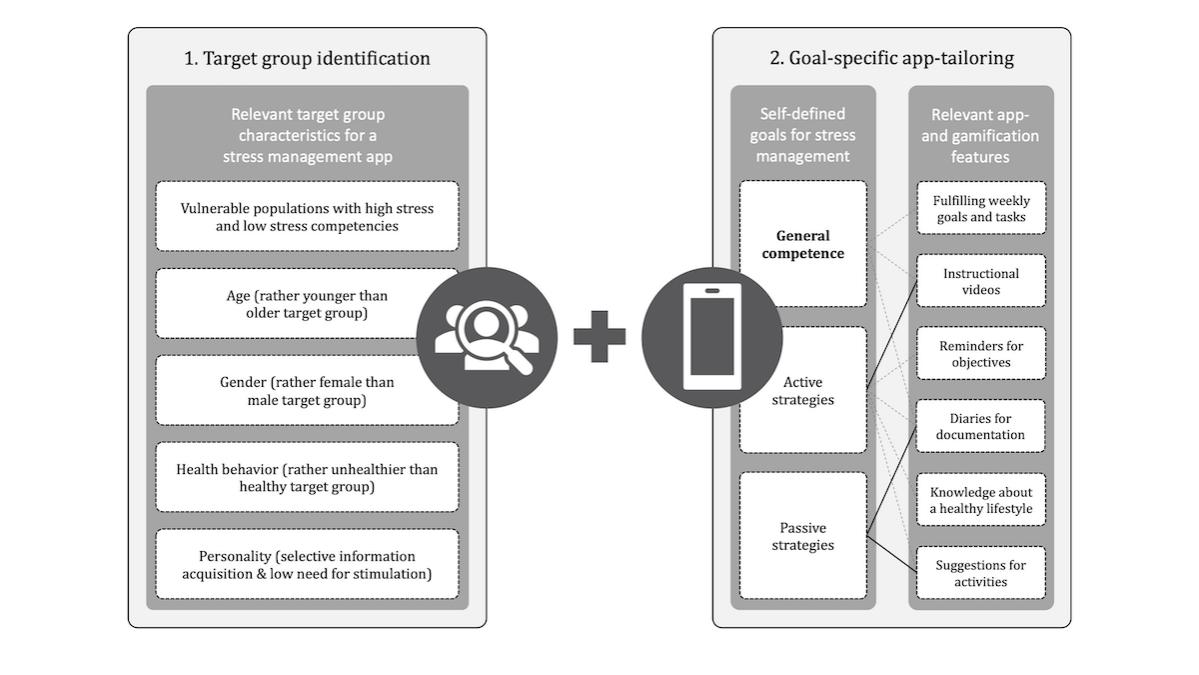
Planned development of a tailored app to increase stress management competencies within families, based on our results. In step 2, the continuous lines depict the significant effects of the calculated linear regression models whilst the dashed lines represent significant correlations.

Nevertheless, there are some important practical implications. Health authorities and mHealth or app developers should take our findings into account when planning and implementing tailored app-based mental health promotion interventions for families. In a first step, target group identification is necessary. The target sample—highly stressed families with low stress competencies, in this case—can be characterized by age, gender, health behavior, and personality. The design of the app, as well as its promotion, should address the unique characteristics of the target group, for example, in this case, a minimalistic user interface that decreases stimulation. In a second step, the app should be designed in such a way that individual setting of a stress management goal is possible. These self-defined goals, defined beforehand, represent specific demands and wishes for relevant app and gamification elements (Figure 2).

### Conclusions

The results of this cross-sectional study show that, in order to develop an app to increase stress management competencies within families, the content should be based on preassessed of competencies, goals, and personality traits of the potential user, and thus, tailored to the user’s needs. Highly stressed parents with low stress management skills want features in an app that make it easier to use and include goal setting techniques. In fact, a need for coaching and instruction was identified, which underpinned prior research showing that guided stress management interventions have more promising results.

This study delivers first results and directions to inform further research in the growing field of mobile and web-based solutions in mental health care. The relationship between integrated elements of behavior change techniques, the usage of gamification elements, and most notably, tailoring of the content of a web-based intervention and the resulting health behavior change show promise that is urgently needed with respect to increasing stress levels and its associated adverse health effects.

## Appendix

Multimedia Appendix 1 Web-based questionnaire on health app tailoring requirements (translated from German).

## Abbreviations

mHealth: mobile health
PSI: Personality System Interaction
