# Supplementary material for: App-Tailoring Requirements to Increase Stress Management Competencies Within Families: Cross-sectional Survey Study
Source: J Med Internet Res. 2021 Jul 30;23(7):e26376. doi: 10.2196/26376 (PMC8367136; doi:10.2196/26376)
Supplement: Multimedia Appendix 1 [file jmir_v23i7e26376_app1.docx]

App-Tailoring Requirements to Increase Stress Management Competencies Within Families: Cross-sectional Survey Study

**Supplementary material**

***Online questionnaire on tailoring requirements of a health app [translated from german].***

**Sociodemographic and Health Variables**

1. How old are you?
2. What gender are you?
   Male/female/diverse
3. How man minutes do you spend doing moderate-intensity activities on a typical day?
   <15/15-30/30-34/45-60/>60
4. How many minutes do you spend doing vigorous-intensity activities per week?
   <25/25-50/50-75/75-100/100-125/125/150/>150
5. How would you rate your dietary behavior?
   Very good/good/uncertain/bad/very bad
6. How many days a week do you have breakfast?
   1/2/3/4/5/6/7
7. How do you rate your ability to unwind and cope with stress?
   Very good/good/uncertain/bad/very bad
8. How do you rate your stress level?
   High/rather high/uncertain/rather low/low

**Personality Variables**

1. A workout and nutrition schedule does not support me in achieving my goals.
   Disagree/mostly disagree/rather disagree/rather agree/mostly agree/agree
2. When I set health goals for myself, I quickly deviate from them.
   Disagree/mostly disagree/rather disagree/rather agree/mostly agree/agree
3. I prefer structures to manage everyday tasks more easily.
   Disagree/mostly disagree/rather disagree/rather agree/mostly agree/agree
4. When I create a workout schedule for myself, I stick to it strictly.
   Disagree/mostly disagree/rather disagree/rather agree/mostly agree/agree
5. When things are not working out the way I want them to, I have a feeling of having to justify myself.
   Disagree/mostly disagree/rather disagree/rather agree/mostly agree/agree
6. I conscientiously pursue my goals.
   Disagree/mostly disagree/rather disagree/rather agree/mostly agree/agree
7. When I experience obstacles in accomplishing my health goals, I consider them a challenge.
   Disagree/mostly disagree/rather disagree/rather agree/mostly agree/agree
8. In terms of my family's health, I am the driving force.
   Disagree/mostly disagree/rather disagree/rather agree/mostly agree/agree
9. I subordinate my own health interests to those of my family.
   Disagree/mostly disagree/rather disagree/rather agree/mostly agree/agree
10. During everyday life, it is important to me to have a lot of personal time for myself.
    Disagree/mostly disagree/rather disagree/rather agree/mostly agree/agree
11. Living together with my family gives me energy to cope with other areas in life.
    Disagree/mostly disagree/rather disagree/rather agree/mostly agree/agree
12. I generally get enthusiastic about spontaneous activities.
    Disagree/mostly disagree/rather disagree/rather agree/mostly agree/agree
13. As a family, we constantly make a conscious decision to engage in health-promoting activities.
    Disagree/mostly disagree/rather disagree/rather agree/mostly agree/agree
14. We barely talk about shared health interests at home.
    Disagree/mostly disagree/rather disagree/rather agree/mostly agree/agree
15. I value intrafamilial communication very much.
    Disagree/mostly disagree/rather disagree/rather agree/mostly agree/agree
16. We frequently engage in family activities during which we interact with each other.
    Disagree/mostly disagree/rather disagree/rather agree/mostly agree/agree

**App-Feature Variables**

1. How relevant do you consider individualization of app content for using a health app?

Totally relevant/relevant/rather relevant/rather irrelevant/irrelevant/totally irrelevant

1. How relevant do you consider fulfilling common weekly goals and tasks for using a health app?
   Totally relevant/relevant/rather relevant/rather irrelevant/irrelevant/totally irrelevant
2. How relevant do you consider connecting the app with wearables for using a health app?
   Totally relevant/relevant/rather relevant/rather irrelevant/irrelevant/totally irrelevant
3. How relevant do you consider increasing knowledge about a healthy lifestyle for using a health app?
   Totally relevant/relevant/rather relevant/rather irrelevant/irrelevant/totally irrelevant
4. How relevant do you consider suggestions for activities with the family for using a health app?
   Totally relevant/relevant/rather relevant/rather irrelevant/irrelevant/totally irrelevant
5. How relevant do you consider diaries for documentation and development of strategies for using a health app?
   Totally relevant/relevant/rather relevant/rather irrelevant/irrelevant/totally irrelevant
6. How relevant do you consider reminders for goals for using a health app?
   Totally relevant/relevant/rather relevant/rather irrelevant/irrelevant/totally irrelevant
7. How relevant do you consider informational or instructional videos for using a health app?
   Totally relevant/relevant/rather relevant/rather irrelevant/irrelevant/totally irrelevant
8. How relevant do you consider an analogue format for children for using a health app?
   Totally relevant/relevant/rather relevant/rather irrelevant/irrelevant/totally irrelevant

**Gamification and feature variables**

Please imagine a health app that includes the following elements. By which element would you be appealed to use a health app?

1. By comparison with others, in a ranking or on a high score list.
   Would appeal to me very much, would appeal to me, would appeal to me rather, would appeal to me rather, would not appeal to me, would not appeal to me at all
2. By controlling and checking my progress.
   Would appeal to me very much, would appeal to me, would appeal to me rather, would appeal to me rather, would not appeal to me, would not appeal to me at all
3. By collecting points for my performance.
   Would appeal to me very much, would appeal to me, would appeal to me rather, would appeal to me rather, would not appeal to me, would not appeal to me at all
4. By collecting shared points with other family members.
   Would appeal to me very much, would appeal to me, would appeal to me rather, would appeal to me rather, would not appeal to me, would not appeal to me at all
5. By receiving awards, recognition, or encouragement.
   Would appeal to me very much, would appeal to me, would appeal to me rather, would appeal to me rather, would not appeal to me, would not appeal to me at all
6. By providing monetary incentives for achieving goals.
   Would appeal to me very much, would appeal to me, would appeal to me rather, would appeal to me rather, would not appeal to me, would not appeal to me at all
7. By linking to the bonus program of the health insurance company.
   Would appeal to me very much, would appeal to me, would appeal to me rather, would appeal to me rather, would not appeal to me, would not appeal to me at all
8. By designing an avatar.
   Would appeal to me very much, would appeal to me, would appeal to me rather, would appeal to me rather, would not appeal to me, would not appeal to me at all
9. By completing tasks under time pressure, e.g., a countdown.
   Would appeal to me very much, would appeal to me, would appeal to me rather, would appeal to me rather, would not appeal to me, would not appeal to me at all
10. By advancing to another level or increasing the level of difficulty.
    Would appeal to me very much, would appeal to me, would appeal to me rather, would appeal to me rather, would not appeal to me, would not appeal to me at all
11. By sharing and comparing my achieved goals with others.
    Would appeal to me very much, would appeal to me, would appeal to me rather, would appeal to me rather, would not appeal to me, would not appeal to me at all
12. By an accompanying storyline.
    Would appeal to me very much, would appeal to me, would appeal to me rather, would appeal to me rather, would not appeal to me, would not appeal to me at all
13. By receiving auditory, haptic or visual feedback.
    Would appeal to me very much, would appeal to me, would appeal to me rather, would appeal to me rather, would not appeal to me, would not appeal to me at all
14. By rating other family members.
    Would appeal to me very much, would appeal to me, would appeal to me rather, would appeal to me rather, would not appeal to me, would not appeal to me at all

**Personally defined goals of interest for stress management**

Which health goals and interventions related to relaxation and coping with stress would be of interest to you?

1. Performance of meditation exercises
   Of interest/not of interest
2. Performance of breathing exercises
   Of interest/not of interest
3. Performance of yoga exercises
   Of interest/not of interest
4. Performance of mindfulness exercises
   Of interest/not of interest
5. Performance of relaxation exercises
   Of interest/not of interest
6. Improvement of my stress management competencies
   Of interest/not of interest
7. Improvement of the ability to perform stress management techniques from anywhere
   Of interest/not of interest
8. Improvement of my personal resilience to stress
   Of interest/not of interest
9. Spending time in nature
   Of interest/not of interest
10. Benefit from wellness and sauna offers
    Of interest/not of interest
